# Detection of sympathetic denervation defects in Fabry disease by hybrid [^11^C]*meta*-hydroxyephedrine positron emission tomography and cardiac magnetic resonance

**DOI:** 10.1007/s12350-023-03205-7

**Published:** 2023-02-28

**Authors:** Constantin Gatterer, Tim Wollenweber, Verena Pichler, Chrysoula Vraka, Gere Sunder-Plassmann, Max Lenz, Christian Hengstenberg, Marcus Hacker, Christian Loewe, Senta Graf, Dietrich Beitzke

**Affiliations:** 1 Division of Cardiology, Department of Medicine II, Medical University of Vienna, Waehringer Guertel 18-20, 1090 Vienna, Austria; 2 Department of Biomedical Imaging and Image-Guided Therapy, Medical University of Vienna, Waehringer Guertel 18-20, 1090 Vienna, Austria; 3 Division of Pharmaceutical Chemistry, Department of Pharmaceutical Sciences, Faculty of Life Sciences, University of Vienna, Vienna, Austria; 4 Division of Nephrology and Dialysis, Department of Medicine III, Medical University of Vienna, Vienna, Austria

**Keywords:** Fabry disease, hybrid imaging, sympathetic denervation, cardiomyopathy, [^11^C]*m*HED

## Abstract

**Background:**

Myocardial glycosphingolipid accumulation in patients with Fabry disease (FD) causes biochemical and structural changes. This study aimed to investigate sympathetic innervation in FD using hybrid cardiac positron emission tomography (PET)/magnetic resonance imaging (MRI).

**Methods and results:**

Patients with different stages of Fabry disease were prospectively enrolled to undergo routine CMR at 1.5T, followed by 3T hybrid cardiac PET/MRI with [^11^C]meta-hydroxyephedrine ([11C]mHED). Fourteen patients with either no evidence of cardiac involvement (*n* = 5), evidence of left ventricular hypertrophy (LVH) (*n* = 3), or evidence of LVH and fibrosis via late gadolinium enhancement (LGE) (*n* = 6) were analyzed. Compared to patients without LVH, patients with LVH or LVH and LGE had lower median T1 relaxation times (ms) at 1.5 T (1007 vs. 889 vs. 941 ms, *p* = 0.003) and 3T (1290 vs. 1172 vs. 1184 *p* = .014). Myocardial denervation ([11C]mHED retention < 7%·min) was prevalent only in patients with fibrosis, where a total of 16 denervated segments was found in two patients. The respective area of denervation exceeded the area of LGE in both patients (24% vs. 36% and 4% vs. 32%). However, sympathetic innervation defects ([11C]mHED retention ≤ 9%·min) occurred in all study groups. Furthermore, a reduced sympathetic innervation correlated with an increased left ventricular mass (*p* = .034, rs = − 0.57) and a reduced global longitudinal strain (GLS) (*p* = 0.023, rs = − 0.6).

**Conclusion:**

Hybrid cardiac PET/MR with [11C]mHED revealed sympathetic innervation defects, accompanied by impaired GLS, in early stages of Fabry disease. However, denervation is only present in patients with advanced stages of FD showing fibrosis on CMR.

**Supplementary Information:**

The online version contains supplementary material available at 10.1007/s12350-023-03205-7.

## Introduction

Fabry disease (FD), a lysosomal storage disorder characterized by a lack of the enzyme alpha-galactosidase A due to different variants of the *GLA* gene, typically leads to structural changes within the cells and tissues of affected organs, including the heart, the kidneys, and the nervous system. These changes are the consequence of glycosphingolipid accumulation, mainly of globotriaosylceramide (Gb3), and subsequent processes.^1^

In patients with classic FD and late-onset cardiac phenotypes, hypertrophy of cardiomyocytes, as well as necrosis and apoptosis, can be found in histological examinations.^2^ Replacement fibrosis of the myocardium constitutes the irreversible organ damage and leads to various clinical complications, including arrhythmia and heart failure.^3^ The clinical signs of FD-associated cardiomyopathy are detectable by electrocardiography (ECG), echocardiography, and cardiac magnetic resonance (CMR) and can be found in up to 60% of male and 50% of female patients.^4^

Modern imaging techniques, such as CMR, including T1 and T2 mapping and late gadolinium enhancement (LGE), facilitate tissue characterization of the myocardium. However, essential aspects of the links between structural and functional changes may be missed. Myocardial scintigraphy, using single-photon emission-computed tomography (SPECT) and positron emission tomography (PET), has already delivered valuable information about coronary microvascular dysfunction, acute and chronic inflammation, and sympathetic cardiac innervation in various diseases, including FD.^5–12^ Cardiac PET/MRI, in particular, may offer further perspectives on the detection of cardiac changes during the course of the disease, combining the simultaneous information of both methods.

As the progressive glycosphingolipid accumulation in FD can ultimately lead to myocardial fibrosis and its subsequent link to cardiac sympathetic denervation, recent studies have evaluated the extent of such accumulation and consequences using [^123^I]-metaiodobenzylguanidine (MIBG) SPECT, and have found evidence that denervated areas might affect left ventricular function in patients with or without LGE.^5,7,11,13^ Furthermore, evidence has previously been found that sympathetic neurons appear more vulnerable than myocardial cells.^14^ However, the direct association of reduced sympathetic innervation with myocardial fibrosis in FD is still unclear. Recently, [^11^C]meta-hydroxyephedrine ([^11^C]*m*HED) hybrid cardiac PET/MRI was proposed as a potential tool to precisely examine the cardiac sympathetic innervation in Fabry disease.^15^

This pilot study, therefore, aimed to demonstrate the feasibility of hybrid cardiac PET/MRI with [^11^C]*m*HED in FD cardiomyopathy and to provide further insights into the presence of myocardial areas with impaired sympathetic innervation or denervated areas and potentially linked abnormalities of routine CMR in patients with different stages of FD.

## Methods

### Patients

The study has been approved by the institutional Ethics Advisory Committee (IRB Nr.: 1562/2018) and complies with the declaration of Helsinki and its amendments. All subjects signed an informed consent form. Between September 2018 and October 2020, patients older than 18 years with genetically confirmed *GLA* variants were prospectively screened for eligibility to undergo hybrid cardiac PET/MRI during their routine baseline or follow-up examination. Patients were assigned to a study group according to their type of cardiac disease manifestation. Contraindications for inclusion in the study were the presence of non-MR-conditional pacemakers, pregnancy, claustrophobia or known contrast agent allergy, and severe kidney disease (eGFR < 30 mL·min/1.73 m^2^). Routine CMR and hybrid cardiac PET/MRI examinations were performed on the same day.

Prior to imaging, patients were instructed to avoid caffeine and nicotine intake for 24 hours before the hybrid cardiac PET/MRI examination. One patient was excluded retrospectively due to the consumption of different medications that could have interfered with the presynaptic sympathetic nervous system.

### Study Groups

Patients were pre-assigned to three different groups according to cardiac involvement in Fabry disease, based on the results from the last yearly routine care echocardiographic (interventricular septal thickness ≥ 12 mm = inclusion definition of LVH) and CMR (presence or absence of myocardial LGE) examinations: **Group LVH-LGE-**: patients with neither left ventricular hypertrophy (LVH) nor myocardial fibrosis, detected by LGE; **Group LVH+LGE-**: patients with LVH only; and **Group LVH+LGE+**: patients with LVH and myocardial fibrosis, reflecting the most advanced stage. For the final group assignment, based on the hybrid cardiac PET/MRI, left ventricular hypertrophy was defined as an end-diastolic left ventricular mass index (LVMI) of > 85 g·m^2^ in male and > 68 g·m^2^ in female patients.^16^

### Hybrid Cardiac PET/MRI

For hybrid cardiac PET/MRI examinations, the Siemens Biograph mMR 3T PET/MRI system (Siemens Healthineers, Erlangen, Germany) was used. Technical details have been described previously.^17^ The protocol included balanced steady-state free precession and LGE imaging in short axis, as well as in two-, three-, and four-chamber views. Pre- and postcontrast T1 mapping was performed to calculate extracellular volume (ECV) in basal, mid-cavity, and apical short-axis slices.

A mean of 347 ± 40 MBq [11C]mHED was administered intravenously with start of the PET acquisition in list mode for a total of 40 minutes. List mode data were then reconstructed to static images (from minute 10 to minute 40, after the initial drop of blood pool activity) and dynamic images (14 frames: 6 × 30, 2 × 60, 2 × 150, 2 × 300, 2 × 600 seconds). The simultaneously recorded ECG was used for synchronizing PET and CMR data acquisition with the cardiac cycle. As a contrast agent for LGE and post contrast T1 mapping 0.15 mL·kg bodyweight gadobutrol (Gadovist, Bayer, Berlin, Germany) was injected.

### CMR

Additional to hybrid cardiac PET/MRI, 1.5T CMR examinations were performed as part of the yearly clinical routine follow-up on a Siemens Avanto Fit MRI scanner (Siemens Healthineers AG, Erlangen, Germany) with a standardized multiparametric protocol. Details are described within the Supplemental Materials.

### Image Analysis

CMR image analysis was done via a dedicated CMR postprocessing software (Medis Suite MR, Medis Medical Imaging, Leiden, Netherlands) by one observer, who was blinded to clinical and PET data. Left ventricular size and function were derived from the 3T cine-balanced steady-state free precession short-axis images at the time of end-diastole and end-systole. The full width at half maximum technique with semi-automatic contouring was performed for the quantification of LGE. The extent of LGE was calculated as percent volume of the total myocardial mass. Segments with any evidence of LGE, after exclusion of artifacts during visual image analysis, were defined as LGE positive, otherwise as LGE negative. ECV was calculated with pre- and post contrast T1 maps from the 3T CMR images using the standard formula and the patient's current hematocrit.^18^ Global longitudinal strain (GLS) was calculated by feature tracking in 2-, 3-, and 4-chamber SSFP cine sequences. Ejection fraction (EF) was calculated using the biplane Simpson's method.

PET images were post-processed by a different observer than for CMR using a dedicated quantification software (MunichHeart, Technical University of Munich, Munich, Germany). The observer was blinded to clinical and CMR data. After defining the long axis, a volumetric sampling algorithm created a polar map of the tracer distribution throughout the left ventricle.^19^

The [^11^C]-mHED retention index (%·min) in the left ventricle was quantified from dynamic images and calculated by dividing tissue activity between the minutes 30 and 40 through the integral of blood counts from minute 0 to minute 40.^20^ Attenuation-corrected images were iteratively reconstructed (three iterations, 21 subsets) in a 256 × 256 matrix. A 17-segment model was used for regional analysis, where the mean retention index was calculated for each segment. Below a retention index of 7%·min, the myocardium was defined as denervated in accordance with previous studies.^21,22^ Although no normal ranges for only mildly impaired sympathetic innervation were published so far, previous studies provided data for healthy control groups who presented with a retention index above 9%·min.^23,24^ Hence, the sympathetic innervation of segments with a retention index of ≤ 9%·min was considered to be impaired.

The analysis of each myocardial segment within the AHA 17-segment model was done for LGE, T1/T2 mapping, ECV, and [^11^C]*m*HED retention. The apical segment was excluded due to a high rate of artifacts.

### Laboratory Markers

Plasma levels of high-sensitive troponin T, NT-proBNP, and creatinine were assessed as part of the routine care within a month before or after the PET/MRI examination. The estimated glomerular filtration rate was calculated using the Chronic Kidney Disease Epidemiology Collaboration equation (CKD-EPI).^25^

### Statistical Analysis

Statistical analysis was performed using SPSS Statistics 28 (IBM, Armonk, New York, USA). Continuous variables were reported as mean ± standard deviation if normally distributed, otherwise as median, and interquartile range. Categorical variables were described as counts and percentages. Differences between the study groups were examined via the Kruskal–Wallis test. In case of significant group differences, multiple testing by analysis of variance was performed, and the Bonferroni correction was applied. Furthermore, Spearman's rank correlation was calculated for the laboratory markers and the variables of routine CMR and hybrid cardiac PET/MRI. A two-tailed *p* value ≤ .05 was considered statistically significant.

## Results

### Patients

A total of sixteen patients (62.5% female, 37.5% male) were enrolled in this study. Due to a failed tracer synthesis, the complete dataset was only available for 14 of the 16 patients, who were recruited. Patient characteristics for the respective groups are displayed in Table 1. The median age was 46.5 years (range, 19–66 years). Five patients matched the criteria for the LVH-LGE- group, three for the LVH+LGE- group, and six for the LVH+LGE+ group. All patients stayed within the same group of cardiac involvement after re-assignment based on the study CMR. The two patients, who were excluded from the analysis were one male and one female patient of the LVH+LGE- group.

**Table 1:** Patient characteristics and results from PET/MRI and laboratory marker

Group^†^:	LVH-LGE- *n* = 5	LVH+LGE- *n* = 3	LVH+LGE+ *n* = 6	*p* value for group differences	*p* value for group differences (multiple testing)
Gender	4 female (80%) 1 male (20%)	1 female (33.3%) 2 male (66.6%)	4 female (67%) 2 male (33%)		
Specific therapy	5 no (100%)	2 ERT (66.6%) 1 Chaperon (33.3%)	2 no (33.3%) 2 ERT (33.3%) 2 Chaperon (33.3%)		
Age (years)	23 [21−25]	33 [26−49]	56.5 [51−60]	0.011	LVH-LGE- vs. LVH+LGE+ : 0.008
Weight (kg)	75 [72−87]	83 [53−89]	81 [62−95]	n.s	
Height (cm)	165 [162−165]	175 [163−185]	168 [164−174]	n.s	
Body surface area (m^2^)	1.88 [1.79−1.98]	1.89 [1.61−2.13]	1.94 [1.68−2.11]	n.s	
LVMI (g·m^2^)	58.4 [55.5−68.5]	86.0 [79.6−87.4]	118 [80.5−153]	0.035	n.s
Max. wall thickness (mm)	8.96 [6.89−9.19]	12.1 [10.9−14.2]	15.4 [14.8−18.7]	0.01	LVH-LGE- vs. LVH+LGE+ : 0.008
LV trabecular mass, end-diastolic (g)	31.3 [29.5−31.5]	43.9 [37.0−48.2]	52.6 [44.4−90.6]	0.025	LVH-LGE- vs. LVH+LGE+ : 0.02
LVEDVI (mL·m^2^)	58.4 [55.5−68.5]	86.0 [79.6−87.4]	118 [80.5−152.7]	n.s	
EF (%)	66.8 [63.4−70.5]	68.0 [67.7−81.6]	76.6 [72.8−78.7]	n.s	
GLS (%)	− 25.4 [− 26.5 to − 25.4]	− 25.5 [− 25.6 to − 21.3]	− 24.2 [− 31.4 to − 18.8]	n.s	
LGE (% of LV mass)	0 [0−0]	0 [0−0]	6.57 [4.09−11.6]	0.003	LVH+LGE+ vs. LVH-LGE-: 0.007 LVH+LGE-: 0.0026
Native 3T T1 (ms)	1290 [1269−1359]	1172 [1146−1257]	1184 [1182−1241]	0.014	LVH-LGE- vs. LVH+LGE+ : 0.028
ECV (%)	32 [28.8−33.4]	24.9 [24.6−25.2]	27.9 [26.6−33.1]	n.s	
[^11^C]*m*HED retention (%·min)	9.68 [8.76−10.71]	10 [9−11]	8.65 [7.11−8.88]	n.s	
Total number of segments	80	48	96		
Denervated segments*	0 (0)	0 (0)	16*** (16.6%)		
Segments with impaired innervation**	29 (36.3%)	8 (16.7%)	65 (67.7%)		
Segments with LGE	0 (0)	0 (0)	31*** (32.2%)		
eGFR (mL·min/1.73 m^2^)	95.2 [90.4−121]	115 [108−121]	98.2 [77,7−109]	n.s	
Hs Troponin T (ng·L)	4.5 [4−8.5]	5 [5−14]	25.5 [22−35]	0.009	LVH-LGE- vs. LVH+LGE+ : 0.011
NT-proBNP (pg·mL)	27.4 [19−28.1]	19.5 [14.3−148]	306 [127−2019]	0.017	LVH-LGE- vs. LVH+LGE+ : 0.023

Values are expressed as median [interquartile range] or absolute numbers (percentage). *p* values are given for group differences

^†^Patient groups: *LVH-LGE-* patients without LVH or LGE, *LVH+LGE-* patients with LVH but without LGE, *LVH+LGE+* patients with LVH and LGE

*Denervation is defined as a [11C]mHED retention of < 7%/min

**Impaired sympathetic innervation is defined as a [11C]mHED retention of ≤ 9%/min

***Total number of affected segments in the LVH+LGE+ group

*ECV* extracellular volume, *ERT* Enzyme replacement therapy, *EF* ejection fraction, *GLS* global longitudinal strain, *LV* left ventricular, *LGE* late gadolinium enhancement, *LVEDVI* left ventricular diastolic volume (indexed), *LVH* left ventricular hypertrophy, *LVMI* left ventricular mass index, *eGFR* estimated glomerular filtration rate, *Hs* high sensitive, *NT-proBNP* N-terminal brain natriuretic peptide, *n.s.* not significant

Seven patients were on a specific therapy, of whom four received enzyme replacement therapy, and three received the pharmacological chaperone Migalastat (Amicus therapeutics).

### Results from Hybrid Cardiac [^11^C]***m***HED PET/MRI

The median LVMI, maximum end-diastolic wall thickness, and the end-diastolic trabecular mass in the LVH-LGE- group were significantly lower than in the LVH+LGE- and LVH+LGE+ groups. Additionally, native T1 values of 3T CMR were lower in patients with LVH. However, left ventricular function, i.e., EF and GLS as well as ECV did not differ between the study groups.

While none of the LVH-LGE- or LVH+LGE- patients had denervated areas, two patients of the LVH+LGE+ group, presented with an individual denervated area of 36% and 32%. The percentual denervated area of the myocardium also correlated with the percentual amount of LGE (*r*_s_ = 0.529; *p* = 0.035), although the area of denervation exceeded the extent of LGE in both affected patients (36% vs. 24% and 32% vs. 4%). Segments with at least mildly impaired sympathetic innervation could be detected in four out of five patients in the LVH-LGE- group, two out of three in the LVH+LGE- group and five out of six in the LVH+LGE+ group, considering a normal [^11^C]*m*HED retention of > 9%·min.

**Figure 1:**
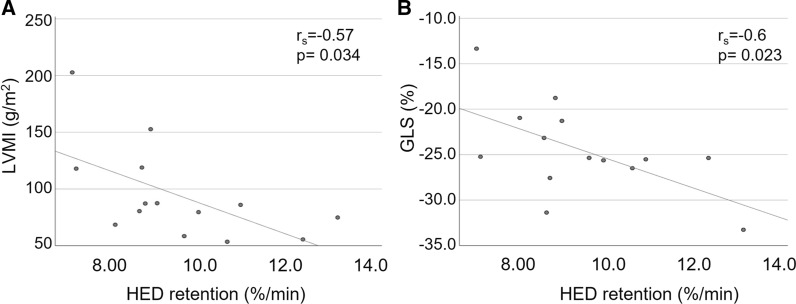
Overview of [11C]mHED retention correlations with LVMI (**a**) and GLS (**b**)

**Figure 2:**
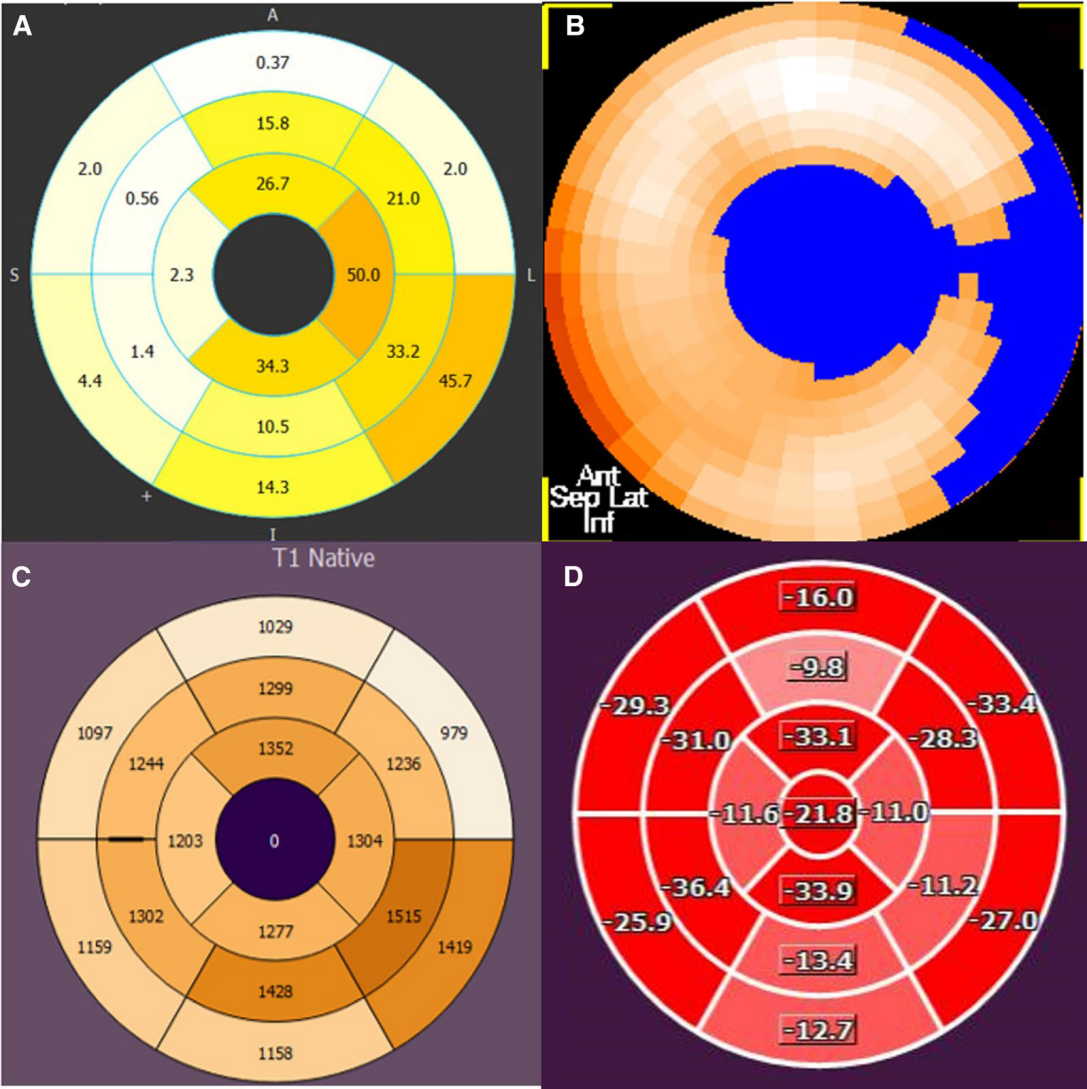
Polar maps of a patient's (LVH+LGE+) 3T CMR late enhancement (**a**). [^11^C]mHED PET (**b**). 1.5T T1 times (**c**). and global longitudinal strain (**d**)

**Figure 3:**
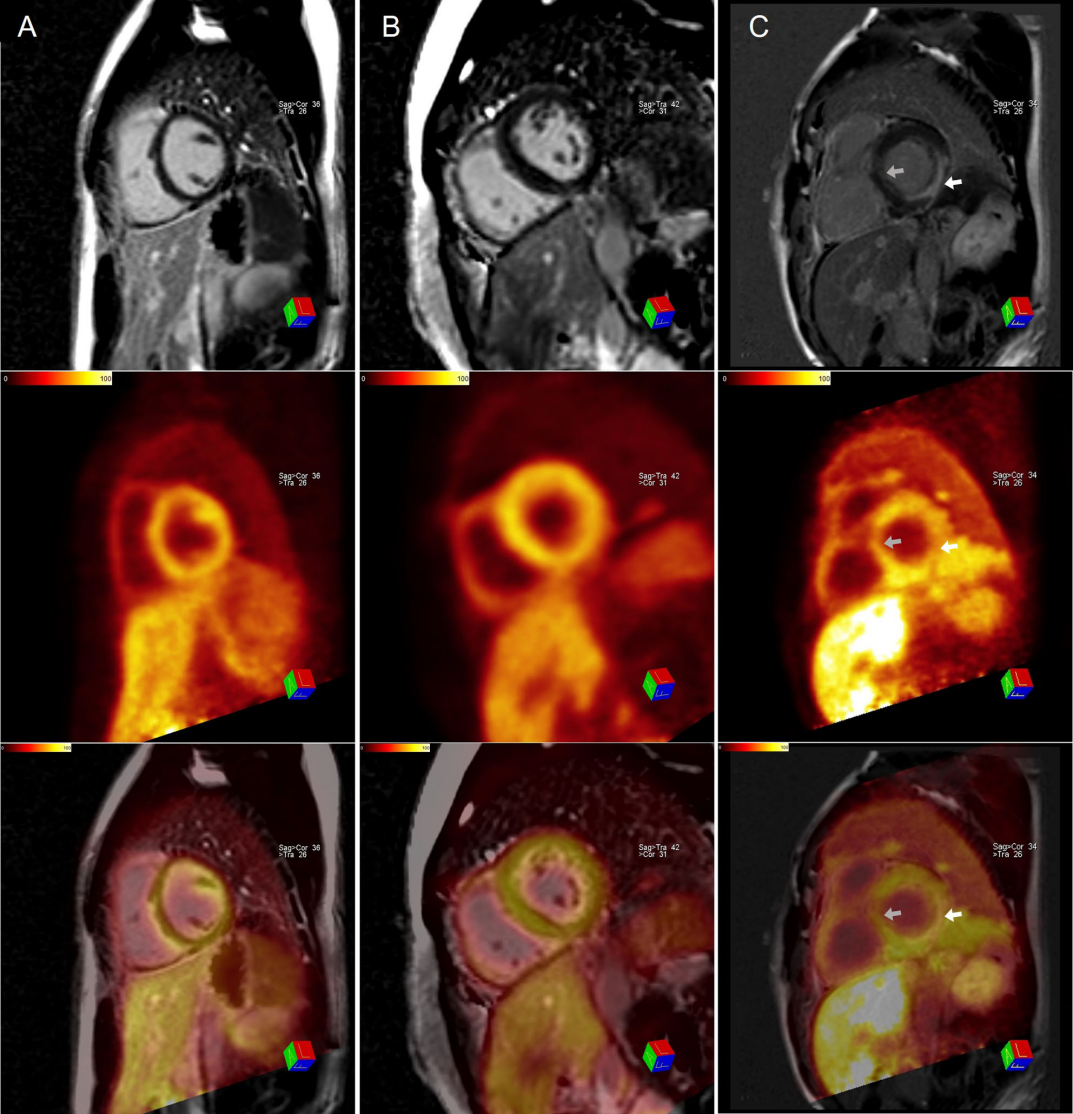
Short-axis views of CMR with late Gadolinium enhancement (first row), the corresponding [11C]mHED retention (second row) and fused hybrid cardiac PET/MRI images (third row) from patients of the LVH-LGE- (**a**) LVH+LGE- (**b**) and the LVH+LGE+ group (**c**). Sympathetic denervation can be found in Patient C in the posterolateral wall (white arrow), corresponding with the area of LGE, but also septal (gray arrow), where no LGE could be detected. Segments with mildly impaired sympathetic innervation could also be found in patients A and B although they cannot be detected by visual analysis

A higher LVMI or maximum wall thickness was associated with a lower [^11^C]*m*HED retention. Furthermore, lower values of [^11^C]*m*HED retention were related to a worse GLS. Figure 1 represents the correlations between sympathetic innervation and parameters that can be obtained during of routine care CMR. Exemplary images of CMR and hybrid cardiac PET/MRI can be found in Figs. 2 and 3.

**Figure 4:**
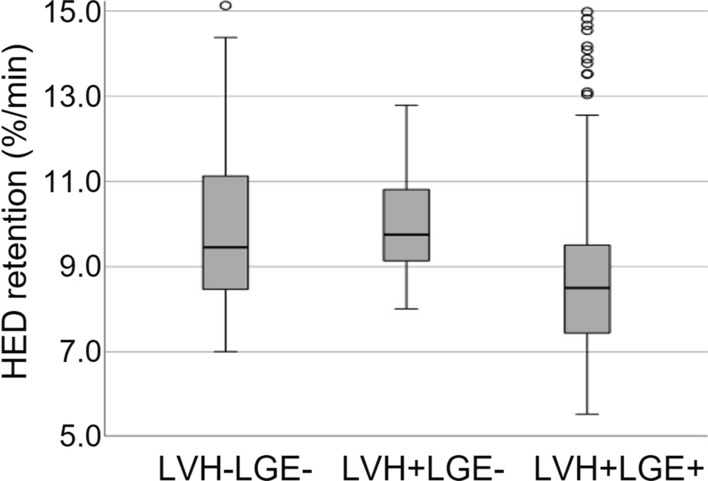
Group comparison of segmental [11C]mHED retention. Patient groups: LVH-LGE-: patients without LVH or LGE; LVH+LGE-: patients with LVH but without LGE; LVH+LGE+ : ptients with LVH and LGE

On a segmental level, [^11^C]*m*HED retention was lower in the segments of LVH+LGE+ patients than in LVH+LGE- or LVH-LGE- patients, as illustrated in Fig. 4. In the LVH+LGE+ group, a total of 16 segments fulfilled the criteria of denervation, whereas 31 segments showed at least minimal signs of LGE. LGE was primarily located within the typical regions of the mid-myocardial segments of the infero-lateral wall, thus, allowing a differentiation from other etiologies of myocardial fibrosis such as ischemic- and other non-ischemic cardiomyopathies.

Results from 1.5T CMR can be found in the Supplemental Materials.

### Laboratory markers

The different stages of disease severity were also reflected by laboratory markers. An overview of the correlations between laboratory and imaging markers can be found in Table 2. Although the glomerular filtration rate did not differ between the study groups, high-sensitive Troponin T and NT-proBNP were higher in the LVH+LGE+ group compared to patients without cardiac involvement. Furthermore, significant correlations could be found between the levels of NT-proBNP and age, maximum wall thickness, the extent of LGE, and 3T T1 values. Comparable correlations could be found for the levels of Troponin T, as high levels were also associated with higher levels of NT-proBNP. No correlations could be found between the laboratory markers and ECV or [^11^C]*m*HED retention.

**Table 2:** Spearman's rank correlation coefficient for laboratory- and imaging markers

	Troponin *T* (ng·L)	NT-proBNP (pg·mL)	eGFR (mL·min/1.73 m^2^)
LVMI (g·m^2^)	0.681*	0.626*	− 0.095
Max. wall thickness (mm)	0.747**	0.622*	− 0.187
LV trabecular mass. end-diastolic (g)	0.722**	0.516	− 0.253
LVEDVI (mL·/m^2^)	− 0.210	− 0.165	0.332
EF (%)	0.321	0.169	0.055
GLS (%)	0.274	0.349	− 0.051
LGE (% of LV mass)	0.824**	0.699**	− 0.329
Native 3T T1 (ms)	− 0.596*	− 0.588*	− 0.093
ECV (%)	0.000	0.333	− 0.224
[^11^C]*m*HED retention (%/min)	− 0.315	− 0.367	0.059

*p ≤ .05

**p ≤ .01

*ECV* extracellular volume, *EF* ejection fraction, *GLS* global longitudinal strain, *LV* left ventricular, *LGE* late gadolinium enhancement, *LVEDVI* left ventricular diastolic volume (indexed), *LVMI* left ventricular mass index, *eGFR* estimated glomerular filtration rate, *Hs* high-sensitive, *NT-proBNP* N-terminal brain natriuretic peptide

## Discussion

To the best of our knowledge, this is the first simultaneous investigation of myocardial structure, function, and sympathetic innervation using hybrid cardiac PET/MRI with [^11^C]*m*HED in patients with FD since previous research on this topic was based on MIBG SPECT. Our study shows that sympathetic denervation, found by [^11^C]*m*HED hybrid cardiac PET/MRI, occurs within the myocardium of patients with advanced cardiac involvement of FD. In addition, areas of sympathetic denervation exceeded those of positive LGE, reflecting the presence of fibrosis. Furthermore, we found segments with impaired sympathetic innervation within all study groups supporting the hypothesis that sympathetic neuronal damage precedes the formation of fibrosis during the process of myocardial glycosphingolipid accumulation in patients with FD.^5^

Moreover, we found a significant correlation between left ventricular mass and [^11^C]*m*HED retention. Indeed, left ventricular hypertrophy reflects a major sign of long-term myocardial glycosphingolipid accumulation in FD.^26^ In our study, a higher LVMI was associated with a worse sympathetic innervation. However, [^11^C]*m*HED did not differ between patients with our without LVH. This might be caused by a continuous process of sympathetic innervation impairment during disease progression, already before the presence of LVH.

Additionally, a recent meta-analysis highlighted that LVH was associated with a reduction of 3T and 1.5T T1 values, reflecting glycosphingolipid accumulation, in patients with FD. As both, 3T and 1.5T CMR systems are regularly used by different hospitals, our study underlined that this hallmark of cardiac involvement in FD can be reliably detected by both scanner field strengths as a higher maximum wall thickness or trabecular mass were associated with lower 1.5T and 3T T1 values. Furthermore, a higher extracellular volume was associated with a higher end-diastolic volume which may be the effect of myocardial fibrosis leading to ventricular dilatation in advanced stages of the disease.^27^ Contrarily to LVMI, no correlations of sympathetic innervation and parameters of myocardial tissue texture imaging with T1/T2 mapping or ECV could be found.

An impairment of sympathetic innervation was previously reported for the heterogeneous group of HFpEF patients, as the authors demonstrated an association of contractile dysfunction, fibrotic burden, and sympathetic denervation.^28^ In FD, the mechanisms related to glycosphingolipid storage within the cells could primarily affect adrenergic function. Compared to patients with ischemic cardiomyopathy, who were studied in the PAREPET trial, it appears that the extent of sympathetic denervation is less in patients with Fabry disease.^29^ This might result from the relatively low scar transmurality in FD compared to ischemic cardiomyopathy. However, [^11^C]*m*HED retention was significantly lower in the segments of FD patients who presented with positive LGE. Sympathetic denervation was linked to a worse prognosis with respect to cardiac events such as arrhythmia and sudden cardiac death in patient with ischemic heart disease and patients who received an implanted cardioverter defibrillator.^29–31^ Indeed, cardiovascular death and in particular sudden cardiac death are also the leading causes of death in patients with FD and might be associated with sympathetic denervation and LGE.^32,33^ Thus, a knowledge of the presence of sympathetic denervation and innervation defects is of high importance in each patient with Fabry disease.

Overall, sympathetic innervation defects are already found in patients without LVH or LGE, although denervation seems to occur primarily in advanced stages of the disease and, as such, might be seen as a prognostic marker. Therefore, patients with positive LGE might be followed closely and considered for specific forms of anti-arrhythmic therapy as this patient group might be at higher risk for cardiac events.^30^ This additional risk should be addressed by future studies.

Although previous studies proved GLS to be an early marker of cardiac dysfunction in FD, we found a normal left ventricular function, monitored by GLS and EF, throughout the three study groups, without significant differences between the groups.^34^ This might be a consequence of the sample size and the heterogenous phenotypes of FD, since both, a normal or impaired GLS, could be found in particular patients within each study group. However, patients with worse sympathetic innervation, measured by [11C]*m*HED retention, also had a worse GLS. This trend is in line with the results of a recent SPECT study, where a link between cardiac MIBG uptake and an impaired longitudinal strain in the respective segments could be detected.^7^ Further research is needed to investigate the clinical effects of sympathetic innervation defects in patients with Fabry disease and the reason of particular patients not developing myocardial innervation defects despite the presence myocardial fibrosis.

Additionally, novel 18F-labeled cardiac radiotracers have been developed recently. They may lead to further improvement in the detection of an impaired cardiac sympathetic innervation as they can be used without an on-site cyclotron and provide more favorable kinetics for the quantification of regional cardiac sympathetic nerve density. ^35–37^

The results of cardiac laboratory biomarkers undermined the advanced disease severity of the LVH+LGE+ group, where higher levels of high-sensitive Troponin T and NT-proBNP could be observed, compared to the LVH-LGE- group. Additionally, we found parallels between the laboratory and imaging markers, as a correlation between both Troponin T and NT-proBNP and the maximum wall thickness, LVMI, the extent of LGE and the values of 3T native T1 mapping could be observed. In contrast to values of native 3T T1 mapping, 1.5T CMR showed no significant correlations with cardiac laboratory markers. However, this may also be a result of the low sample size since 3T and 1.5T T1 mapping correlated well. The accuracy of high-sensitive Troponin T and NT-proBNP for the detection of cardiac involvement in Fabry disease were demonstrated in previous studies.^38,39^ Nevertheless, a combination of imaging and laboratory markers appears to be useful for the detailed characterization of organ involvement in Fabry disease.

Since FD is a rare disease, our study offers insights into only a small cohort of patients, thus, representing a limitation of our study. By selecting patients at different disease stages, we aimed to compensate for this issue. Although our center has extensive experience with the production of [^11^C]*m*HED, synthesis failures could not be prevented, thus, leading to the exclusion of individual patients for the respective analysis. Cut-offs for [^11^C]*m*HED retention are based on previous studies evaluating transplanted hearts and healthy control groups; however, the lack of a healthy control group constitutes another limitation of our study.

## Conclusion

Although PET/MRI will remain an investigational tool due to high costs and the need for an on-site cyclotron, hybrid cardiac [^11^C]*m*HED PET/MRI delivers additional insights into the course of Fabry disease. Impaired sympathetic innervation, occurring before the onset of LVH or LGE and correlating with left ventricular mass and left ventricular function, might pose an additional risk for affected patients, thus, highlighting [^11^C]*m*HED retention as a potential risk marker for cardiac events. Overall, our data might help to shed some light on additional useful cardiac imaging techniques in addition to echocardiography and CMR and their potential to evaluate as-yet-undetected myocardial damage and associated risks for these patients. Further studies will be needed to analyze the long-term clinical impact of myocardial denervation in patients with FD.

## New Knowledge Gained

[^11^C]*m*HED PET/MRI facilitates the detection of sympathetic impairment in patients with FD even before the onset of left ventricular hypertrophy or fibrosis. However, sympathetic denervation could only be found within the myocardium of patients with late gadolinium enhancement. A reduced sympathetic innervation correlates with left ventricular mass and with an impaired left ventricular function, measured by global longitudinal strain (GLS).

### Supplementary Information

Below is the link to the electronic supplementary material.

Supplementary file1 (DOCX 19 kb)

Supplementary file2 (PPTX 5138 kb)

Supplementary file3 (MP3 5601 kb)

## Data Availability

The data underlying this article are available in the article.

## Abbreviations

FD: Fabry disease
ECG: Electrocardiogram
CMR: Cardiac magnetic resonance
LGE: Late Gadolinium Enhancement
SPECT: Single-photon emission-computed tomography
PET: Positron emission tomography
[^11^C]*m*HED: [^11^C]meta-hydroxyephedrine
LVH: Left ventricular hypertrophy
EF: Left ventricular ejection fraction
GLS: Global longitudinal strain
LVMI: Left ventricular mass index
