# Supplementary material for: Detection of sympathetic denervation defects in Fabry disease by hybrid [11C]meta-hydroxyephedrine positron emission tomography and cardiac magnetic resonance
Source: J Nucl Cardiol. 2023 Feb 28;30(5):1810–21. doi: 10.1007/s12350-023-03205-7 (PMC10558396; doi:10.1007/s12350-023-03205-7)
Supplement: Supplementary file 1 — Supplementary file1 (DOCX 19 kb) [file 12350_2023_3205_MOESM1_ESM.docx]

SUPPLEMENTAL MATERIALS

Detection of sympathetic denervation defects in Fabry disease by hybrid **[^11^C]*meta*-hydroxyephedrine positron emission tomography and cardiac magnetic resonance**

**Methods: CMR**

The purpose of this second CMR was to identify and enroll patients for the current study and to maintain the comparability for field strength dependent parameters, such as T1 and T2 values, between the present, previous and future examinations, that were and will be routinely performed on the 1.5 vender. Another purpose was to detect possible alterations in 1.5T T1 and T2 mapping that go in parallel with sympathetic denervation and should therefore be evaluated during the routine CMR follow-ups. The protocol included an examination of left ventricular structure and function, as well as advanced tissue characterization by three short axis slices and T1 and T2 mapping, based on current recommendations by the Society of Cardiovascular Magnetic Resonance.^1^

**Results from 1.5T CMR**

Similar to 3T Hybrid PET/MRI, median native 1.5T T1 values were significantly lower in patients with LVH. 1.5T T2 values did not differ between the study groups.

**Correlations of hybrid cardiac** **[^11^C]*m*HED PET/MRI with routine 1.5T CMR**

While no association between the sympathetic innervation and the values of ECV or T1-/T2-mapping could be drawn, T1-values were found to be lower in patients with a higher left ventricular wall thickness or a higher trabecular mass (r_s_-0,596, p=0.025 and r_s_=0.599, p=0.025, respectively). Furthermore, ECV was higher in patients with a higher end-diastolic volume (r_s_=0.673, p=0.033). A significant correlation could also be found between the 3T and 1.5T T1-mapping values (r_s_=0.764, p=0.002).

**Correlation of 1.5T CMR with laboratory markers**

In contrast to 3T T1 values, no correlations were found between the levels of Troponin T, NT-proBNP or any other laboratory marker and values of 1.5T T1- and T2-mapping.

**SUPPLEMENTAL TABLE 1.** Values from 1.5T CMR

| Group^†^: | LVH- LGE- **n=5** | LVH+LGE- **n=3** | LVH+LGE+ **n=6** | p-value for group differences | p-value  for group differences (multiple testing) |
| --- | --- | --- | --- | --- | --- |
| Native 1.5T T1 (ms) | 1007 [984−1021] | 889 [841−937] | 941 [898−965] | 0.027 | LVH-LGE- vs. LVH+LGE-: .03 |
| Native 1.5T T2 (ms) | 50.5 [49.4−52.2] | 45.9 [44.1−48.6] | 50.8 [48.8−53.4] | n.s. |  |
| Values are expressed as median [interquartile range] or absolute numbers (percentage). P-values are given for group differences.  † Patient groups: LVH-LGE-: patients without LVH or LGE; LVH+LGE-: patients with LVH but without LGE; LVH+LGE+: patients with LVH and LGE.  LGE, late gadolinium enhancement; LVH, left ventricular hypertrophy; n.s., not significant. | | | | | |

**SUPPLEMENTAL TABLE 2.** Spearman's rank Correlation coefficient for laboratory- and imaging markers

|  | Troponin T (ng/L) | NT-proBNP (pg/mL) | eGFR  (ml/min/1,73 m²) |
| --- | --- | --- | --- |
| Native 1.5T T1 (ms) | -0.404 | -0.275 | 0.033 |
| Native 1.5T T2 (ms) | 0.091 | 0.345 | -0.385 |
| * p ≤ 0.05  ** p ≤ 0.01  eGFR. estimated glomerular filtration rate; Hs. high-sensitive; NT-proBNP. N-terminal brain natriuretic peptide. | | | |

1. Messroghli DR, Moon JC, Ferreira VM, Grosse-Wortmann L, He T, Kellman P*, et al.* Clinical recommendations for cardiovascular magnetic resonance mapping of T1, T2, T2* and extracellular volume: A consensus statement by the Society for Cardiovascular Magnetic Resonance (SCMR) endorsed by the European Association for Cardiovascular Imagi. J Cardiovasc Magn Reson 2017; **19**.
