# Supplementary material for: Detection of sympathetic denervation defects in Fabry disease by hybrid [11C]meta-hydroxyephedrine positron emission tomography and cardiac magnetic resonance
Source: J Nucl Cardiol. 2023 Feb 28;30(5):1810–21. doi: 10.1007/s12350-023-03205-7 (PMC10558396; doi:10.1007/s12350-023-03205-7)
Supplement: Supplementary file 2 — Supplementary file2 (PPTX 5138 kb) [file 12350_2023_3205_MOESM2_ESM.pptx]

## Slide 1
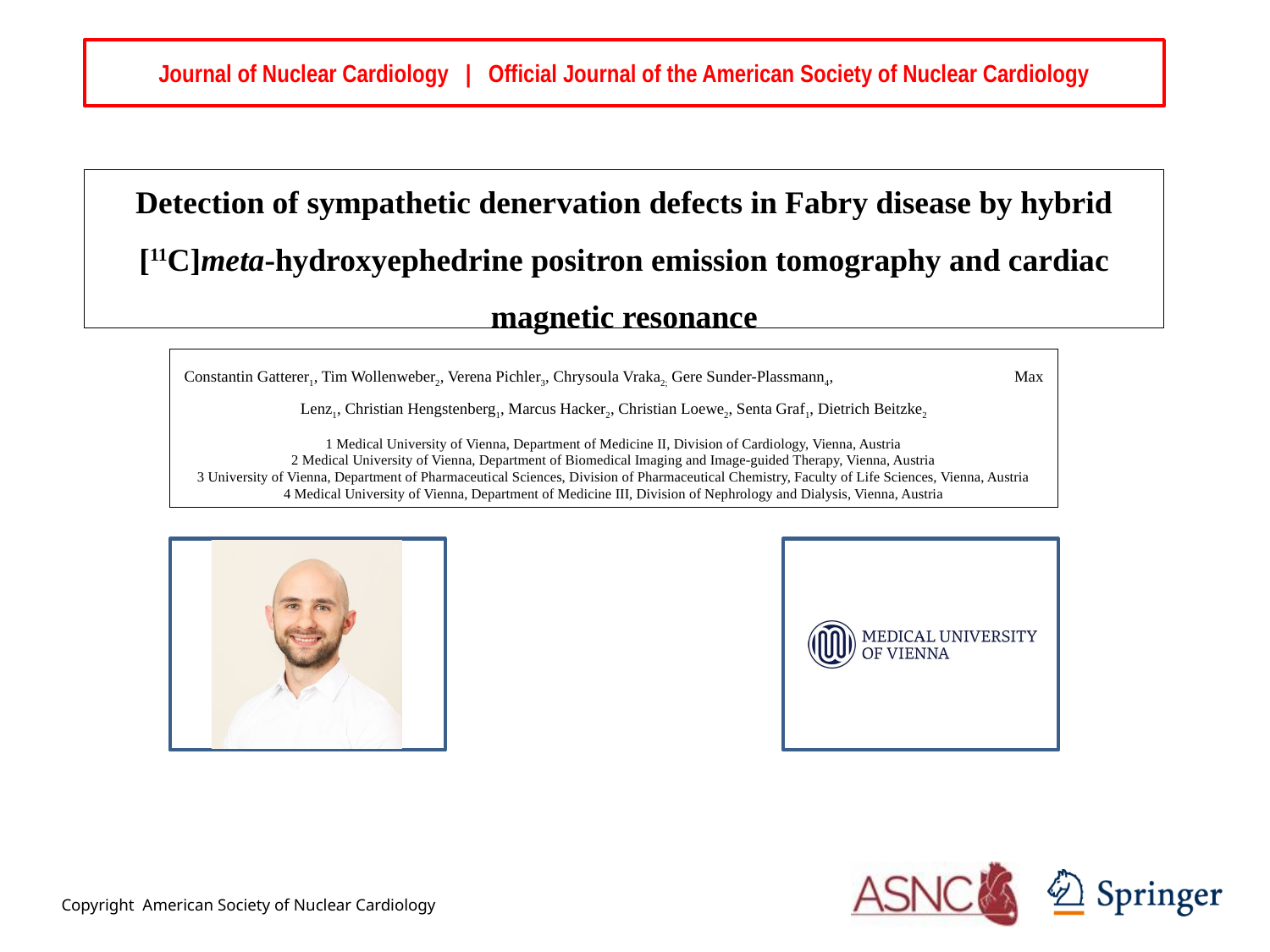

Journal of Nuclear Cardiology | Official Journal of the American Society of Nuclear Cardiology
# Detection of sympathetic denervation defects in Fabry disease by hybrid [11C]meta-hydroxyephedrine positron emission tomography and cardiac magnetic resonance
Constantin Gatterer1, Tim Wollenweber2, Verena Pichler3, Chrysoula Vraka2; Gere Sunder-Plassmann4, Max Lenz1, Christian Hengstenberg1, Marcus Hacker2, Christian Loewe2, Senta Graf1, Dietrich Beitzke2
1 Medical University of Vienna, Department of Medicine II, Division of Cardiology, Vienna, Austria
2 Medical University of Vienna, Department of Biomedical Imaging and Image-guided Therapy, Vienna, Austria
3 University of Vienna, Department of Pharmaceutical Sciences, Division of Pharmaceutical Chemistry, Faculty of Life Sciences, Vienna, Austria
4 Medical University of Vienna, Department of Medicine III, Division of Nephrology and Dialysis, Vienna, Austria
Head shot of author
required
Institution
Picture/Logo
Optional
Copyright American Society of Nuclear Cardiology

## Slide 2
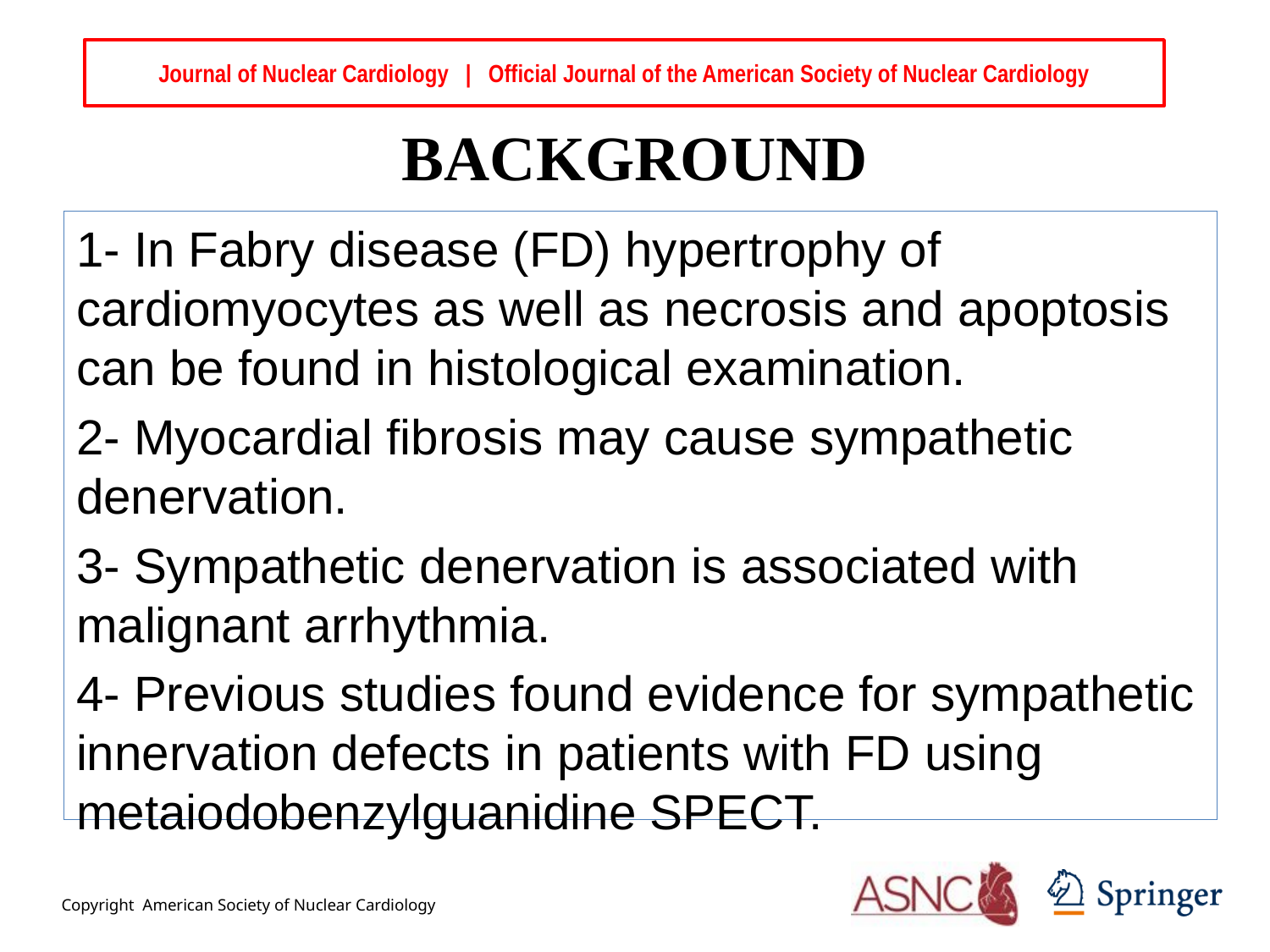

Journal of Nuclear Cardiology | Official Journal of the American Society of Nuclear Cardiology
# BACKGROUND
1- In Fabry disease (FD) hypertrophy of cardiomyocytes as well as necrosis and apoptosis can be found in histological examination.
2- Myocardial fibrosis may cause sympathetic denervation.
3- Sympathetic denervation is associated with malignant arrhythmia.
4- Previous studies found evidence for sympathetic innervation defects in patients with FD using metaiodobenzylguanidine SPECT.
Copyright American Society of Nuclear Cardiology

## Slide 3
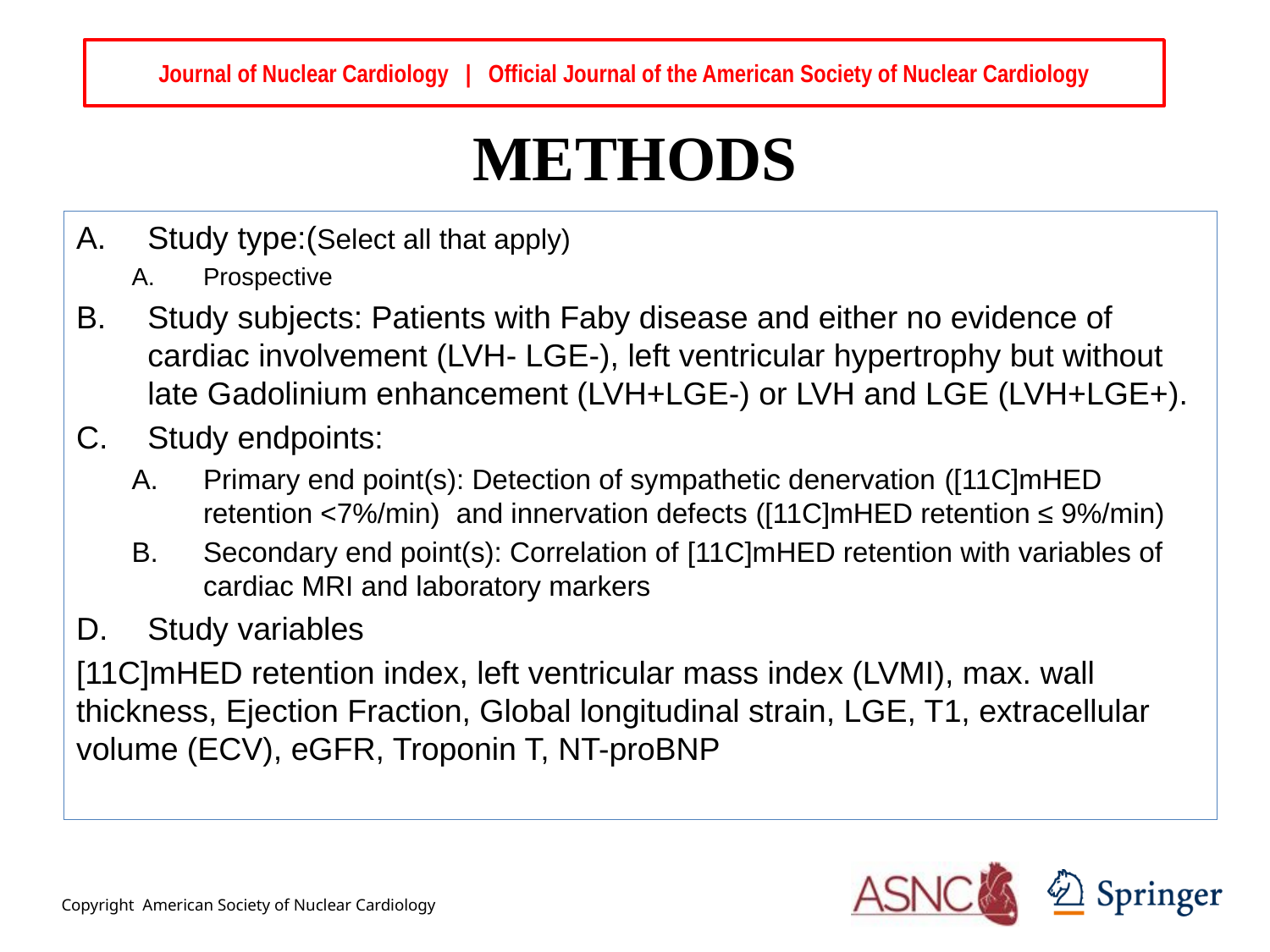

Journal of Nuclear Cardiology | Official Journal of the American Society of Nuclear Cardiology
# METHODS
Study type:(Select all that apply)
Prospective
Study subjects: Patients with Faby disease and either no evidence of cardiac involvement (LVH- LGE-), left ventricular hypertrophy but without late Gadolinium enhancement (LVH+LGE-) or LVH and LGE (LVH+LGE+).
Study endpoints:
Primary end point(s): Detection of sympathetic denervation ([11C]mHED retention <7%/min) and innervation defects ([11C]mHED retention ≤ 9%/min)
Secondary end point(s): Correlation of [11C]mHED retention with variables of cardiac MRI and laboratory markers
Study variables
[11C]mHED retention index, left ventricular mass index (LVMI), max. wall thickness, Ejection Fraction, Global longitudinal strain, LGE, T1, extracellular volume (ECV), eGFR, Troponin T, NT-proBNP
Copyright American Society of Nuclear Cardiology

## Slide 4
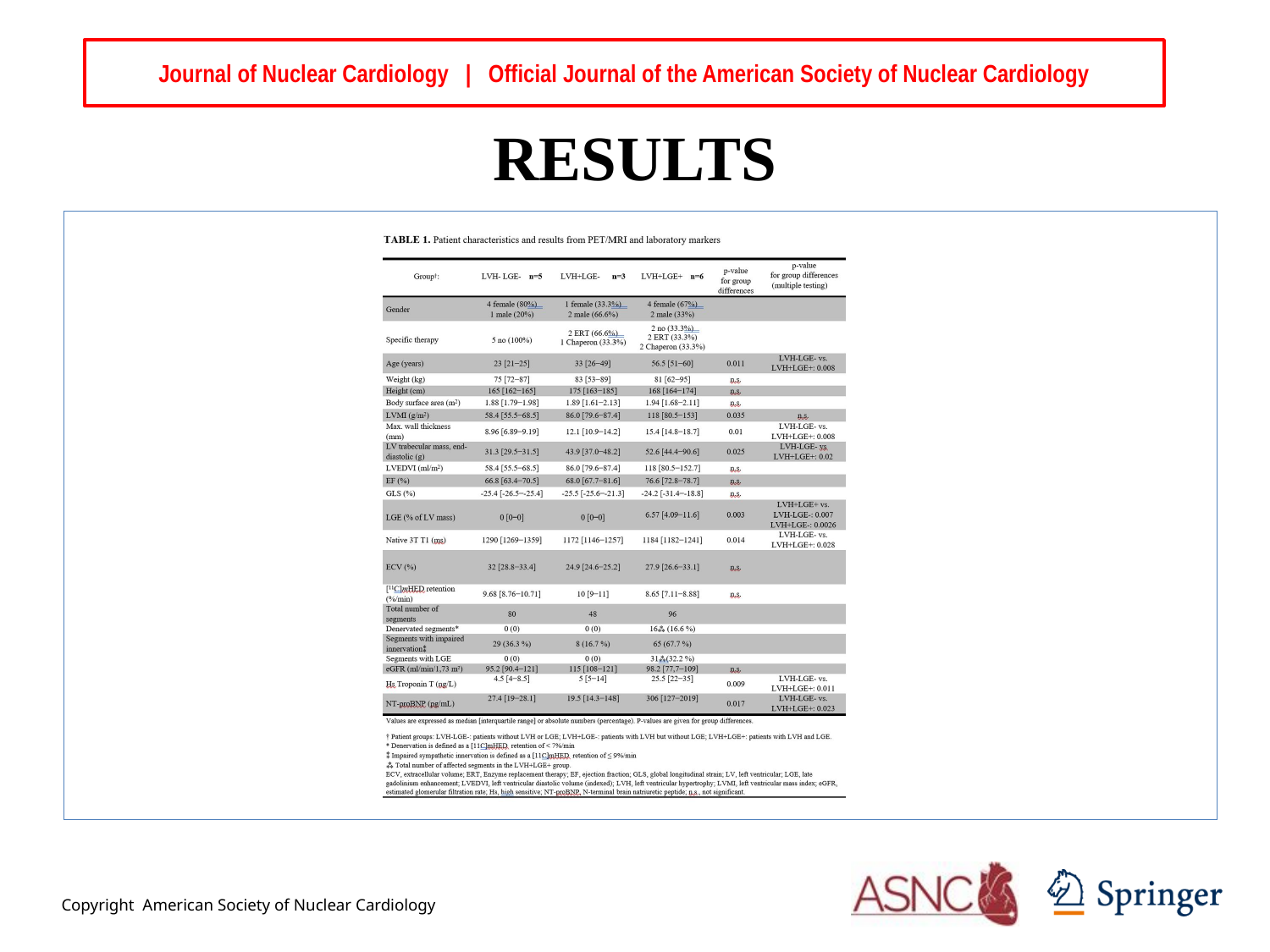

Journal of Nuclear Cardiology | Official Journal of the American Society of Nuclear Cardiology
# RESULTS
Copyright American Society of Nuclear Cardiology

## Slide 5
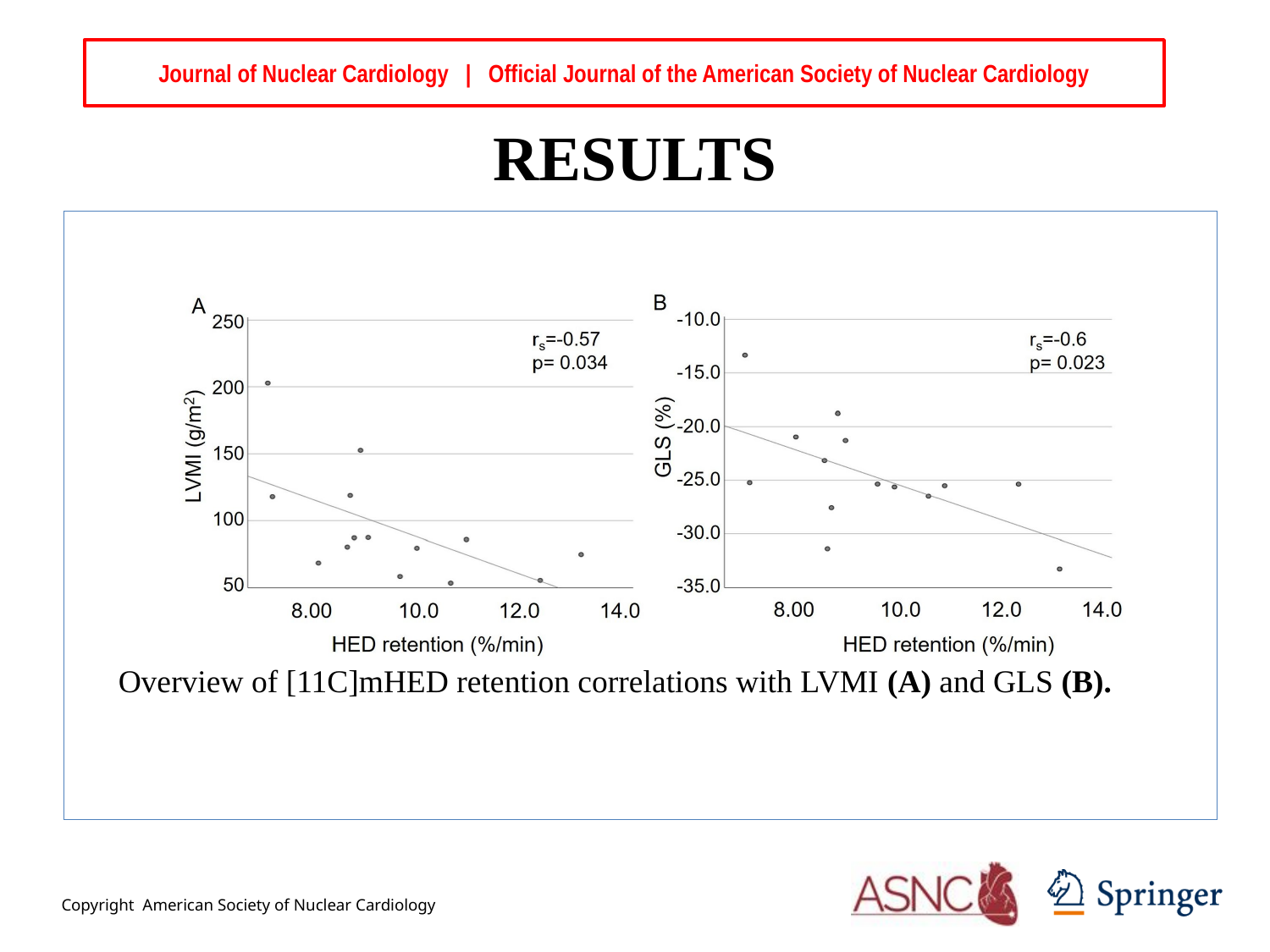

Journal of Nuclear Cardiology | Official Journal of the American Society of Nuclear Cardiology
# RESULTS
Insert a key table or a key figure
If figure, insert legend
Overview of [11C]mHED retention correlations with LVMI (A) and GLS (B).
Copyright American Society of Nuclear Cardiology

## Slide 6
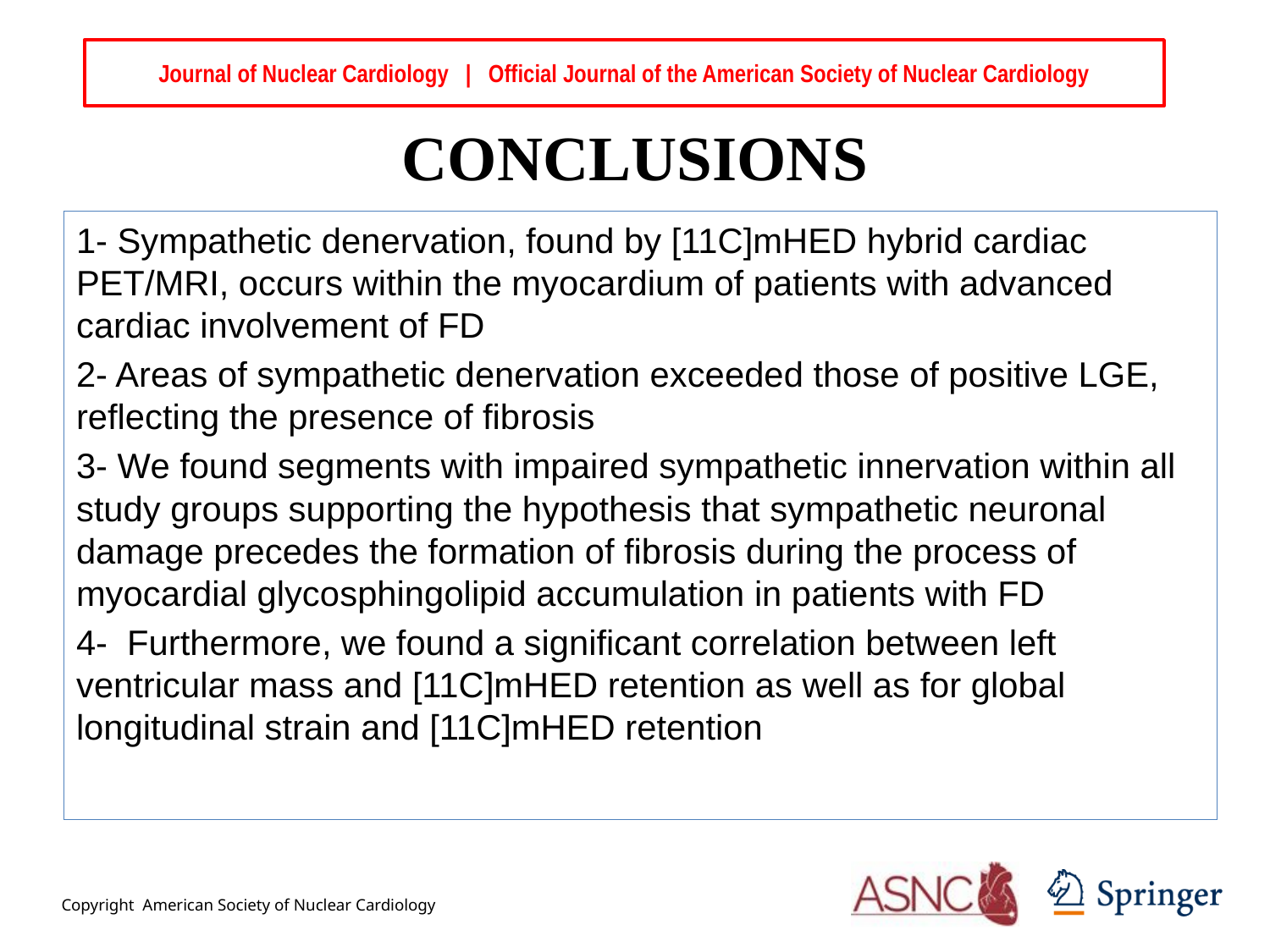

Journal of Nuclear Cardiology | Official Journal of the American Society of Nuclear Cardiology
# CONCLUSIONS
1- Sympathetic denervation, found by [11C]mHED hybrid cardiac PET/MRI, occurs within the myocardium of patients with advanced cardiac involvement of FD
2- Areas of sympathetic denervation exceeded those of positive LGE, reflecting the presence of fibrosis
3- We found segments with impaired sympathetic innervation within all study groups supporting the hypothesis that sympathetic neuronal damage precedes the formation of fibrosis during the process of myocardial glycosphingolipid accumulation in patients with FD
4- Furthermore, we found a significant correlation between left ventricular mass and [11C]mHED retention as well as for global longitudinal strain and [11C]mHED retention
Copyright American Society of Nuclear Cardiology
